# Application of Mixed/Augmented Reality in Interventional Cardiology

**DOI:** 10.3390/jcm13154368

**Published:** 2024-07-26

**Authors:** Mohsen Annabestani, Ali Olyanasab, Bobak Mosadegh

**Affiliations:** 1 Weill Cornell Medicine, Cornell University, New York, NY 10065, USA; 2 Institute for Integrated Circuits, Johannes Kepler University Linz, 4040 Linz, Austria; olyanasab.a@gmail.com

**Keywords:** augmented reality, mixed reality, interventional cardiology

## Abstract

This review explores the transformative applications of augmented reality (AR) and mixed reality (MR) technologies in interventional cardiology. The integration of these cutting-edge systems offers unprecedented potential to enhance visualization, guidance, and outcomes during complex cardiac interventional procedures. This review examines four key domains: (1) medical AR/MR systems and technological foundations; (2) clinical applications across procedures like TAVI, PCI, and electrophysiology mapping; (3) ongoing technology development and validation efforts; and (4) educational and training applications for fostering essential skills. By providing an in-depth analysis of the benefits, challenges, and future directions, this work elucidates the paradigm shift catalyzed by AR and MR in advancing interventional cardiology practices. Through meticulous exploration of technological, clinical, and educational implications, this review underscores the pivotal role of these innovative technologies in optimizing procedural guidance, improving patient outcomes, and driving innovation in cardiovascular care.

## 1. Introduction

The rapid evolution of medical imaging technologies and computational power has catalyzed a paradigm shift in interventional cardiology. The integration of AR and MR systems into cardiac interventional procedures represents a transformative frontier, offering unprecedented potential to enhance visualization, guidance, and, ultimately, patient outcomes [1,2,3]. By seamlessly blending virtual and real-world elements, these cutting-edge technologies empower clinicians with dynamic, multidimensional perspectives and real-time guidance during complex cardiovascular interventions. The application of AR and MR in interventional cardiology spans a diverse array of procedures and modalities. From transcatheter aortic valve implantation (TAVI) [2,3,4] and percutaneous coronary interventions [1,5,6] to electrophysiology mapping and ablation [7,8,9], these technologies have demonstrated remarkable potential for improving procedural accuracy, efficiency, and decision-making. By integrating multiple imaging modalities, such as fluoroscopy, computed tomography (CT), magnetic resonance imaging (MRI), and ultrasound, AR and MR systems provide clinicians with augmented visualization of intricate cardiac anatomy and catheter positioning, overcoming the limitations of traditional 2D imaging [10,11,12]. Furthermore, AR and MR technologies have shown promise in streamlining procedural workflows, reducing reliance on cumbersome standard LCD monitors, and minimizing procedural disruptions [13,14,15]. The hands-free nature of these systems, facilitated by head-mounted displays or holographic projections, enables clinicians to maintain situational awareness and focus on the procedure at hand, potentially enhancing patient safety and outcomes.

Importantly, the applications of AR and MR in interventional cardiology extend beyond the operating room. These technologies have demonstrated significant value in medical education and training, enabling realistic simulations of complex cardiac interventions [16,17,18]. Through immersive and interactive learning experiences, medical students, residents, and practicing clinicians can cultivate essential skills and spatial reasoning abilities, fostering competency and proficiency in interventional cardiology procedures. As with any transformative technology, the integration of AR and MR into interventional cardiology requires rigorous validation, standardization, and continuous refinement. Ongoing efforts are underway to address challenges related to image optimization, system performance, user interface design, and seamless integration with existing clinical workflows [19,20,21]. Additionally, collaborative efforts between clinicians, engineers, and researchers are crucial to unlock the full potential of these technologies and drive innovation in cardiovascular care. By reviewing the applications, benefits, challenges, and future directions of AR and MR in interventional cardiology, this paper aims to provide a holistic understanding of this rapidly evolving field. Through meticulous exploration of the technological foundations, clinical applications, and educational implications, this review underscores the transformative impact of these innovative technologies on procedural guidance, patient outcomes, and the overall advancement of interventional cardiology practices.

**Figure 1:**
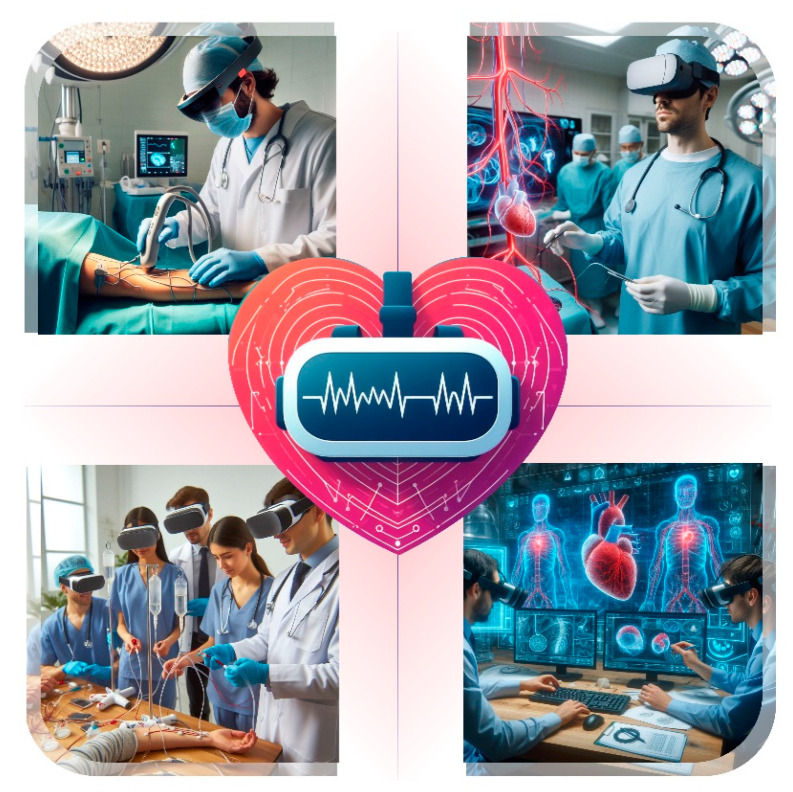
Applications of AR/MR systems in interventional cardiology are divided into four sections: Medical AR/MR systems (**top-right**), Clinical Application and Evaluation of AR/MR Systems (**top-left**), Technology Development and Validation (**bottom-right**), and Educational and Training Applications (**bottom-left**). To draw this figure, we have used Microsoft Copilot.

This paper presents a comprehensive analysis of the application of Augmented/Mixed Reality (AR/MR) in the context of interventional cardiology, delineating the discourse into four primary sections, as illustrated in Figure 1. Initially, an overview is provided regarding various AR/MR systems proposed for medical applications. Subsequently, an examination ensues regarding the practical utilization of such systems in clinical practice, encompassing both individually tailored and commercially available alternatives. Furthermore, the discussion extends to ongoing research endeavors aimed at the development and validation of AR/MR technologies specifically tailored for medical applications. Additionally, this paper explores the role of AR/MR within educational and training domains pertinent to medical professionals. Finally, prospective considerations are presented, elucidating anticipated future developments in the field of AR/MR technology in interventional cardiology, thus offering valuable insights into its forthcoming trajectories and potential advancements. 

## 2. Medical AR/MR Systems

Medical AR/MR systems represent cutting-edge technologies poised to transform the landscape of interventional cardiology. These systems offer a novel approach to visualizing intricate cardiac anatomy and guiding procedures in real-time. By seamlessly integrating multiple imaging modalities, including ultrasound, electrocardiogram, and intravascular ultrasound, they provide clinicians with high-fidelity, real-time views of the patient’s anatomy and procedural guidance. Through the overlay of virtual images onto the physical environment, AR/MR systems empower clinicians to engage with both tangible and virtual realms, enhancing their situational awareness and procedural efficiency. Moreover, research indicates that these systems hold promise for improving procedural outcomes [3], reducing reliance on cumbersome standard LCD monitors [13], and minimizing procedural disruptions [14]. Consequently, AR/MR systems have the potential to augment visualization, procedural guidance, and decision-making in interventional cardiology, ultimately fostering enhanced patient care and outcomes.

Introducing the Enhanced Interaction Electrophysiology Visualization and System (ELVIS), Avari Silva et al. aimed to revolutionize real-time intraprocedural imaging within electrophysiology (EP) laboratories [4]. ELVIS, a fusion of the HoloLens headset and SentEP software, delivers 3D digital images of electroanatomic maps and catheter locations. In a groundbreaking clinical trial, physicians tested ELVIS against the standard system, revealing its remarkable enhancement in navigation accuracy, as demonstrated by Wilcoxon signed-rank testing, which showed an error of 2.99 ± 1.91 mm for ELVIS compared to 4.50 ± 3.74 mm for the standard system (*p* < 0.005). Notably, ELVIS showcases potential in amplifying physician skills and improving patient outcomes, marking a significant advancement in EP laboratory technology. In a parallel endeavor, Chahine et al. delved into the realm of MR technology, exploring its impact on procedural outcomes in cardiac catheterization laboratories. Through the integration of various imaging modalities into an MR head-mounted display (MR-HMD), the study enrolled 50 patients, 33 of whom underwent RHC and 29 had a diagnostic CA performed, compared to 232 patients in the control group, and the result was in that the implementation of MR-HMD was associated with a significantly reduced procedure time of 20 min (IQR 14–30) vs. 25 min (IQR 18–36), *p* = 0.038 [3]. Emphasizing improved procedural efficiency, the study underscores the transformative potential of wireless medical MR-HMD for real-time procedural guidance. Meanwhile, Holland et al. pioneered a virtual reality (VR) blood simulation tool aimed at aiding clinicians during acute cardiac events. By simulating blood flow dynamics post-treatment, the tool provides critical insights unattainable through traditional 2D medical imagery. By leveraging VR and MR technologies, the tool empowers clinicians with enhanced decision-making capabilities, bridging gaps in acute cardiovascular event management [2].

In a different domain, Franson et al. unveiled a proof-of-concept system for real-time online MR visualization of cardiac magnetic resonance images [1]. Employing highly undersampled radial trajectories for rapid acquisition, the system enables real-time 3D MR renderings of magnetic resonance images, fostering advancements in MR-guided interventions and imaging technologies. Pasquali et al. addressed the challenge of preprocedural planning for left atrial appendage (LAA) occlusion using computed tomography (CT) images. Their innovative software, rooted in MR, facilitates real-time 3D visualization and planning, streamlining the process across various LAA morphologies. The morphological analysis of the holographic anatomical models was successfully applied for all four patients, irrespective of their morphology, and was completed in less than 10 min. The study’s success, coupled with the software’s remote collaboration capabilities, signifies a transformative shift in LAA occlusion planning, promising enhanced accuracy and efficiency [5]. 

Further expanding the application of MR, Alonso-Felipe et al. demonstrated the efficacy of MR glasses in assisting physicians during real-time ultrasound-guided femoral arterial cannulation. Their study underscores the transformative potential of MR systems in improving procedural accuracy and reducing intervention time and costs, paving the way for the broader adoption of interventional cardiology procedures [6]. Finally, Salavitabar et al. explored the conversion of 3D rotational angiography (3DRA) into AR models for visualizing congenital heart disease, leveraging the Microsoft HoloLens 2 (HL2) MR headset. Their study highlights the feasibility and benefits of AR models in enhancing visualization and educational capabilities in congenital heart disease management, where visualization and identification of structures were graded as “very easy” in 81.1% (*n* = 73) of AR compared to 67.8% (*n* = 61) of Computed Models (CM) [7]. 

**Figure 2:**
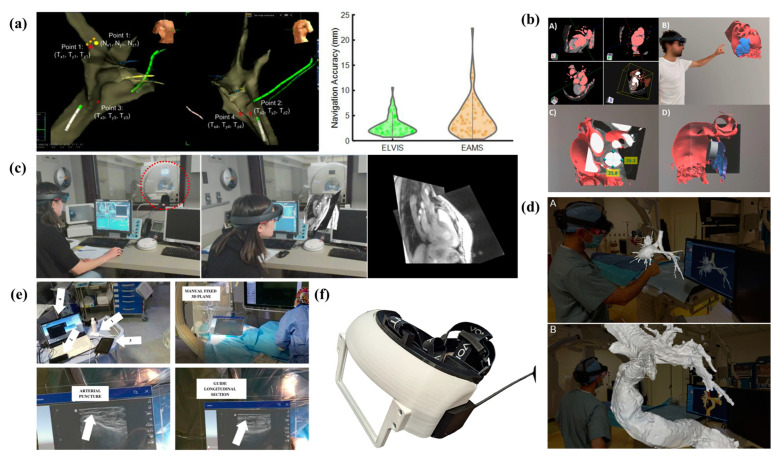
(**a**) (Left) target Point 1 (Tx1, Ty1, Tz1) and the marked catheter location Point 1 (Nx1, Ny1, Nz1) during a first-in-human clinical study. (Right) Navigation accuracy using ELVIS and EAMS [4]. (**b**) Workflow for holographic model generation and analysis involves (A) Creating an endocardial isosurface for the 3D model, (B) Visualizing the model using the MR application, (C) Conducting quantitative morphology analysis to identify the maximum and minimum diameters of the landing zone; (D) virtually implanting the LAA occluder into the holographic model [5]. (**c**) Operation of the complete system at the MR scanner: (Left) A user wearing a mixed-reality headset sits at the scanner control computer, preparing to scan a healthy volunteer inside the scanner bore (outlined with a red dashed circle). (Middle) During the scan, the user observes a real-time multi-slice rendering of the volunteer’s heart. (Right) The user’s view of this rendering [1]. (**d**) Side-by-side comparison of augmented reality and computer models of reconstructed 3DRAs post Hemi-Fontan operation, including airway (A) and a magnified intraluminal view post-complex Fontan palliation (B) [7]. (**e**) Different views of the system: The top-left quadrant displays the main hardware components of the transmitter module: (1) ultrasound scanner, (2) video capture card, (3) tablet, and (4) laptop. The top-right quadrant shows a plane rendering of ultrasound images, manually aligned by the doctor using a button located in the top right corner of the window (next to the close button). The bottom-left quadrant depicts an arterial puncture assisted by the system, while the bottom-right quadrant shows the guide inserted into the artery, viewed from a longitudinal section [6]. (**f**) Mixed reality head-mounted display used in the study [3].

In Figure 2, a representation of various medical AR/MR systems for applications in interventional cardiology is presented.

## 3. Clinical Application and Evaluation of AR/MR Systems

The landscape of interventional cardiology has been reshaped by the rapid integration of AR/MR systems. These cutting-edge technologies offer a paradigm shift in how complex cardiac procedures are visualized, navigated, and executed. By seamlessly merging real-time imaging data with patients’ anatomical structures, AR/MR systems provide clinicians with unprecedented insight and precision during catheter-based interventions. Evaluating the safety, efficacy, and broader impact of these systems is imperative for harnessing their full potential in improving clinical outcomes. As such, AR/MR systems represent a transformative frontier in advancing patient care in interventional cardiology.

In a prospective preclinical study, Bloom et al. examined the feasibility and usability of the MantUS^TM^ (SentiAR, Inc., St. Louis, MO, USA) MR ultrasound system in simulated vascular access scenarios. Focusing on addressing challenges inherent in current ultrasound-guided vascular access procedures, such as suboptimal viewing angles and needle tip visualization, the MantUS^TM^ system showed a reduction in needle repositions and an improvement in access attempt quality compared to conventional ultrasound alone. The implementation of MantUS^TM^ led to a faster time to access (*p* = 0.04), a reduced number of access attempts (*p* = 0.02), and fewer needle repositions (*p* < 0.0001) in comparison to conventional ultrasound. Participants found the system easy to use with enhanced spatial awareness, marking a promising step toward reducing failed access attempts and adverse events in daily procedures. A post-participant survey revealed high levels of usability (87%) and the belief that MantUS^TM^ may reduce adverse outcomes (73%) and failed access attempts (83%). However, the study also highlighted the necessity for future hardware and software enhancements to optimize the user interface and overall system performance, underscoring the need for technical achievements to be coupled with design [8]. Southworth et al. delved into the realm of cardiac electrophysiology, evaluating the performance of an MR system for intraprocedural use. With a focus on real-time visualization and control of clinically relevant data, the study addressed the mental fatigue experienced by interventional physicians during minimally invasive procedures. Through human-in-the-loop testing and observational clinical studies, the authors assessed the system’s hardware performance, image quality, and usability, emphasizing the importance of standardized performance tests and metrics to evaluate MR head-mounted displays. Despite challenges such as rendering electroanatomic maps consistently and polygon counts affecting display performance, the study underscored the potential benefits of MR systems in enhancing procedural accuracy and efficiency in cardiac electrophysiology [9].

Redondo et al. explored the integration of MR technology into interventional cardiology, specifically focusing on transcatheter aortic valve implantation (TAVI) procedures. By utilizing the HoloLens 2 MR headset, the study integrated holograms with echocardiogram imaging in real-time, offering vascular puncture guidance support. Although complexities were identified, such as integrating real-time ultrasound images into anatomical structures, the study concluded that MR technology holds promise in improving the integration of various imaging modalities during cardiovascular procedures, ultimately enhancing patient safety parameters [10]. 

In a pioneering effort, Bruckheimer et al. undertook a feasibility investigation into generating real-time interactive 3D digital holograms within a typical catheterization laboratory setting for clinical medical imaging purposes. The study encompassed eight patients, with five undergoing transcatheter ASD closure utilizing 3DTEE and three being assessed through 3D rotational angiography. The principal aim was to illustrate that the anatomical points of reference identified on standard 3DTEE and 3DRA imaging could be similarly identified using real-time holographic images. Employing the RealView Holographic Display system, researchers produced high-quality, clinically pertinent, 3D real-time color dynamic holograms that were visible with ease and of satisfactory quality under standard cath lab illumination and settings. This system facilitated various interactions, including marking, cropping, zooming/magnification, rotation, slicing, and moving of the hologram. The investigation validated the viability of generating clinically pertinent real-time interactive 3D digital holograms during routine cardiac catheterization procedures utilizing acquired volumetric data. The authors noted that each observer could identify all relevant anatomic landmarks, such as the beating atria, valves, septa, static pulmonary arteries, and coronary arteries, as effortlessly as with a conventional 2D screen from a wide range of angles [11]. Opolski et al. demonstrated the successful use of wearable computers, specifically Google Glass (Google Inc., Mountain View, CA), in computed tomography-guided percutaneous revascularization of a chronically occluded right coronary artery. The wearable device projected 3D computed tomographic reconstructions onto its screen, facilitating visualization of the distal coronary vessel and guidewire advancement direction during the intervention. This innovative application highlights the potential of wearable computers to improve operator comfort and procedural efficiency in interventional cardiology, particularly in chronic total occlusion recanalization attempts [12]. In another study conducted by the same research group, Opolski et al. conducted a single-center prospective pilot study assessing the feasibility and safety of using an AR glass for computed tomography-assisted percutaneous revascularization of coronary chronic total occlusion (CTO PCI). The study utilized wearable AR smart glasses to display computed tomography angiography (CTA) datasets during CTO PCI procedures. Despite the longer procedural time, the AR device facilitated the manipulation of multiple imaging data and led to decreased contrast use. In comparison with standard CTO PCI, CTA-assisted recanalization of CTO using a wearable computer exhibited a higher frequency of selecting the first-choice stiff wire (0% vs. 40%, *p* < 0.001) and reduced contrast exposure (166 ± 52 vs. 134 ± 43 mL, *p* = 0.03). Nevertheless, the overall CTO success rates and safety outcomes remained comparable between both groups. The study suggested potential benefits of antegrade guidewire selection and contrast use reduction compared with standard CTO PCI without CTA overlay. Overall, the authors concluded that AR glass usage during CTO PCI was feasible, safe, and well-received by operators without additional complications [21]. 

**Figure 3:**
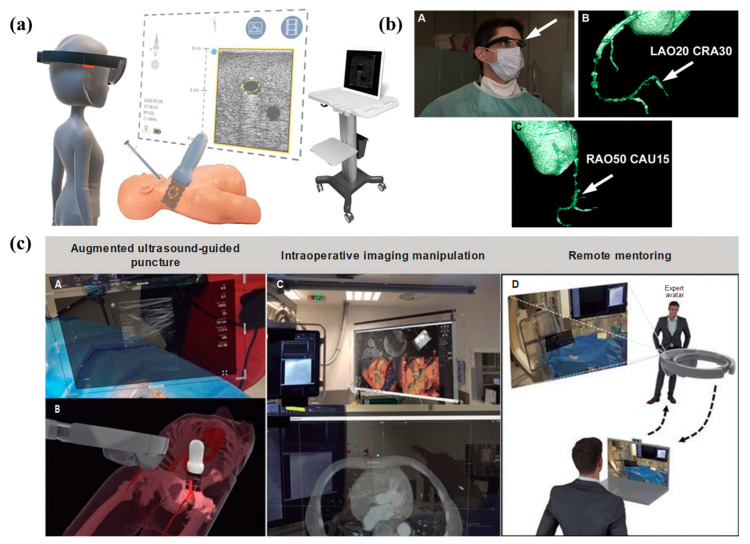
(**a**) Illustration of the MantUS^TM^ MR platform. The image depicts a participant wearing the MR headset, with a standard ultrasound screen available in the room [8]. (**b**) Display of CTA images through Google Glass. (A) A cardiologist observes CTA images on Google Glass during a chronic total occlusion (CTO) recanalization procedure. (B,C) Three-dimensional reconstructions are directly projected onto the Google Glass screen, providing precise visualization of the distal RCA trajectory [12]. (**c**) A: Integration of vascular ultrasound during puncture. B: Schematic representation of simultaneous integration during vascular puncture. C: Image from software analysis of manageable computed tomography scans under aseptic conditions. D: Simulation of remote mentoring [10].

Some of the examples of the proposed systems showcasing the clinical application and evaluation of AR/MR systems are represented in Figure 3.

## 4. Technology Development and Validation

The use of MR technology in interventional cardiology has revolutionized medical procedures by providing real-time visualization and guidance during complex cardiac interventions. This innovative approach combines virtual and AR to enhance the precision and safety of procedures, ultimately improving patient outcomes. The integration of MR technology has significantly impacted the field of interventional cardiology and has the potential to shape the future of cardiovascular care.

In a pioneering study, Bloom et al. compared the efficacy of an MR system, CommandEP^TM^ v1 (SentiAR, Inc., St. Louis, MO, USA), against the conventional electroanatomic mapping system (EAMS) in cardiac electrophysiological testing. The MR system, integrated with a Microsoft HoloLens head-mounted display and proprietary software, provides real-time 3-dimensional cardiac geometry and catheter locations. Dubbed the Cardiac Augmented REality (CARE) study, its goal was to evaluate the MR system’s impact on procedural efficiency and navigational accuracy. Surprisingly, the results indicated no prolongation of case duration with MR system use, with no significant differences observed in navigation times or shell generation. Furthermore, the potential benefits of MR systems, including streamlined communication and enhanced accuracy, were highlighted. The authors stressed the importance of integrating novel technologies into procedural specialties and the necessity of well-crafted simulated environments to mitigate associated risks [13]. In another approach, Linte et al. developed an interventional system tailored for minimally invasive cardiac surgery, allowing therapy delivery within the beating heart without direct vision or radiation exposure. Anchored in a VR environment, the system melds pre-operative data with real-time intra-operative imaging to provide surgical guidance. Pre-operatively, dynamic MR cardiac image datasets generate subject-specific dynamic heart models. Intra-operatively, real-time interventional guidance via transesophageal echocardiography (TEE) is enhanced by a feature-based registration technique mapping pre-operative cardiac models onto intra-operative ultrasound images. Augmenting the virtual surgical environment are real-time ultrasound images and virtual models of magnetically tracked surgical tools displayed on single flat-screen monitors and head-mounted displays. Successfully guiding mitral valve implantations and atrial septal defect (ASD) closure interventions in porcine subjects, this system underscores the feasibility of VR-enhanced ultrasound (US) platforms for minimally invasive procedures [14].

In tandem with these advancements, Liu et al. unveiled an AR system for guiding transcatheter procedures in structural heart disease, addressing the limitations of fluoroscopy. This innovative system provides a 3D visual environment and quantitative feedback on the catheter tip position within the heart, overcoming fluoroscopy’s depth perception and contrast limitations. By segmenting the heart and spine from the patient’s CT or MRI scans, the researchers fabricated a 3D-printed phantom, facilitating in vitro testing of the system. By incorporating standard views and orientations accessible through voice commands, the system represents a significant leap forward. Challenges discussed include incorporating ultrasound for direct imaging of heart tissue and automating spine segmentation in real-time, underscoring the need for further investigation to develop the system for clinical use [15]. Expanding on this concept, Palumbo et al. proposed an AR-based navigation system for radiation-free interventional procedures in interventional cardiology. The study utilized electromagnetic (EM) sensors and QR codes for calibration and tracking, offering a quantitative assessment of both intra- and inter-operators. Each operator conducted 10 evaluation tests to assess variability. Results indicated a mean error of 2.70 ± 0.36 mm and 2.68 ± 0.79 mm for intra- and inter-operator tests, respectively [16].

In a study exploring the frontier of MR technology, Brun et al. delved into the proof of concept and feasibility of using MR holograms of individual 3D heart models for pre-operative diagnostic use in complex congenital heart disease. Employing standard cardiac computed tomography angiograms (CTA) images, a 3D heart model was segmented, aiming to assess hologram quality and explore potential benefits. The study garnered highly positive feedback from congenital heart disease professionals, indicating the potential of MR holograms in revolutionizing pre-operative diagnostics [17]. In another novel approach, Annabestani et al. introduced a machine learning-driven approach for predicting the roll angle of an Intracardiac Echocardiography (ICE) catheter by analyzing bi-plane fluoroscopy images. The objective was to overcome the absence of roll angle sensing capabilities and enable comprehensive 6-DOF tracking for AR/MR systems. Their research involved extracting landmark scalar values from synchronized frames of videos captured from Antero-Posterior (AP) and Left Anterior Oblique at 90 degrees (LAO90) fluoroscopy imaging planes. The model was trained using 360 paired bi-plane fluoroscopic images acquired during simulated procedures in the catheterization lab at the New York Presbyterian Hospital. The outcomes demonstrated the model’s efficacy, exhibiting a minimal error rate and high precision. The Normalized Mean Square Error values were approximately 3 × 10^−3^, with an average error of about 1.25% and Mean Absolute Error values around 4.5 degrees, along with a standard deviation below 5 degrees. These findings suggest that the developed model holds promise for enabling 6-DOF tracking of an ICE catheter, thereby enhancing the accuracy of real-time guidance in AR/MR applications [22].

**Figure 4:**
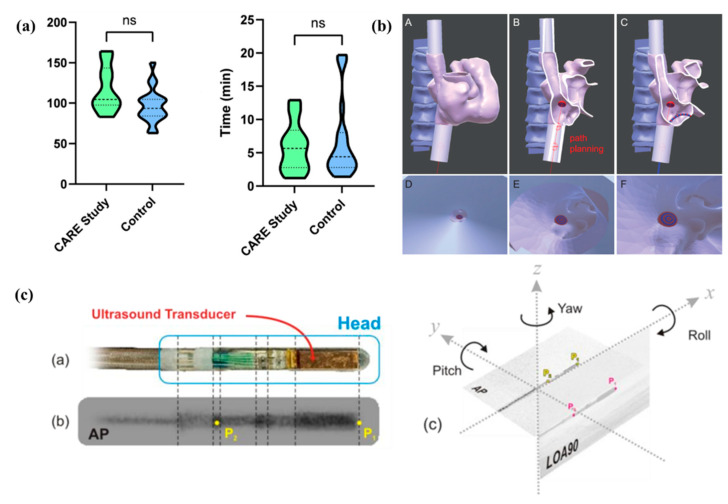
(**a**) Case duration and fluoroscopy time compared: Violin plots depict the CARE patient cohort (green) versus the control cohort (blue). No statistically significant difference was observed in either case duration or fluoroscopy time. The dashed line represents the mean, while the dotted lines show the 25th and 75th percentiles. (ns = not significant) [13]. (**b**) Enhanced visualization on an augmented reality device: A: Heart and spine 3D rendering displayed as holograms on HoloLens. B: Preprocedural planning shown with red lines indicating the ideal transseptal puncture site (target rings). C: Catheter rendered in 3D space; position determined from fluoroscopic images. D–F: Virtual camera attached to the catheter endpoint provides a first-person view during insertion through the inferior vena cava (D), entering the right atrium (E), and approaching the transseptal puncture target (F) [15]. (**c**) Roll Angle Prediction for Intracardiac Echocardiography Catheter during bi-plane fluoroscopy: (a) Sensor head of the ICE catheter, (b) Fluoroscopy image of the ICE catheter sensor head, (c) 3D representation of AP and LAO90 planes [22].

Figure 4 shows examples of technology development and validation of AR/MR systems for applications in interventional cardiology.

## 5. Educational and Training Applications

Incorporating MR technology into educational curricula and training programs presents a compelling opportunity to enhance the competency and proficiency of healthcare professionals specializing in interventional cardiology. MR, encompassing both VR and AR, has emerged as a dynamic tool in this field, offering innovative avenues for educational and training initiatives. By providing immersive and interactive learning experiences, MR technology enables learners to delve into complex cardiac anatomy and pathology, interact with lifelike 3D models of the heart and vasculature, and simulate interventional procedures in a controlled, risk-free environment. This approach fosters a deeper understanding of disease processes, facilitates skill development, and cultivates spatial reasoning abilities among medical students, residents, and practicing physicians. Research indicates that MR-based educational applications not only augment comprehension and retention but also contribute significantly to the cultivation of competent and adept interventional cardiologists [20].

Continuing the trajectory of innovation in interventional cardiology, Jang et al. introduced the Smart Glasses Cannula Guide System, a breakthrough designed to streamline cannula insertion by displaying blood vessels in real-time. Through smart wearable glasses, AR images extracted from angiogram CT scans are projected and aligned seamlessly with CT images using fiducial markers. The system’s development involved integrating CT and geometric markers alongside a phantom demonstrating varying material densities of blood vessels, muscles, and fats. Experimentation in the Ubuntu operating system environment, utilizing a camera and OpenCV library while connecting smart glasses to the Android platform environment, yielded promising results. By facilitating the task of venous puncture at the onset of intervention by projecting deep veins, this system shows the potential for enhancing procedural efficiency, echoing successful commercial implementations in related fields [18]. Meanwhile, Fagan et al. expanded upon these advancements with a review of 3D imaging technologies in guiding complex catheterization procedures for congenital heart disease (CHD). Discussing the utility of rotational angiography (RA) and 3-dimensional rotational angiography (3DRA), the authors delve into imaging techniques, contrast injection protocols, and the integration of AR devices like EchoNavigator. Despite highlighting advantages, such as high-resolution imaging, challenges still persist, including image optimization and suitability for specific lesions. Looking forward, the paper explores rapid cine angiographic acquisition protocols and C-arm CT applications, underscoring ongoing efforts to refine and broaden the scope of these technologies in CHD interventions [19].

In parallel, Torabinia et al. proposed a deep learning-driven tracking method to quantitatively track mock cardiac interventions on custom 3D-printed heart phantoms. By leveraging bi-plane fluoroscopic imaging and external fiducial markers, this method ensures continuous catheter positioning in a unified coordinate system. With a U-Net model for segmentation tasks, the proposed architecture achieved reliable segmentation scores, promising enhanced precision for minimally invasive cardiovascular surgery. Notably, the study introduced a hybrid training simulator for structural heart disease interventions, signaling a shift toward more data-driven, technology-integrated training methodologies in the cardiovascular domain [20]. In another approach, Opolski et al. presented a compelling case study demonstrating the application of AR technology in guiding leadless transcatheter pacemaker implantation in a patient with complex cardiac anatomy. Utilizing computed tomography angiography (CTA) and Google Glass, the team navigated the challenges posed by the patient’s altered fluoroscopic orientation, optimizing lead positioning within the subpulmonic ventricle. This case exemplifies the invaluable role of AR in enhancing procedural precision and efficacy, particularly in navigating complex anatomical variations during interventional procedures [23].

**Figure 5:**
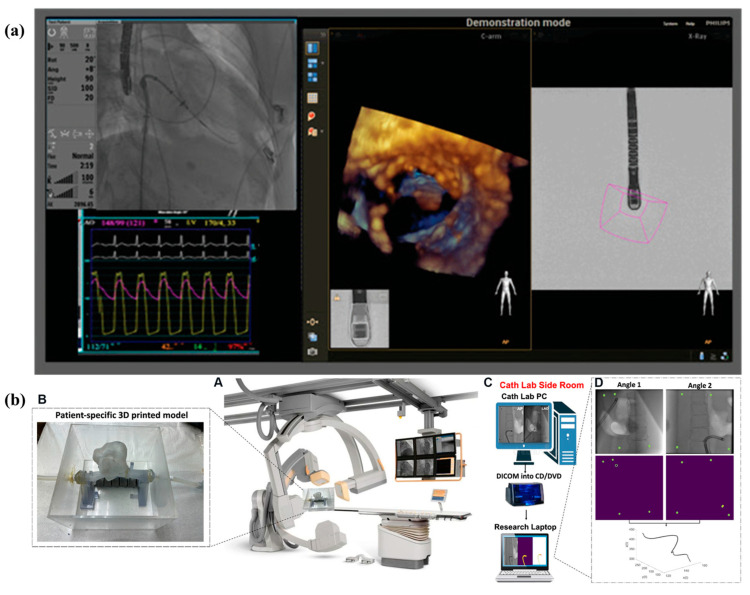
(**a**) Images from EchoNavigator, which are used to guide mitral valve interventions. The transesophageal echocardiography (TEE) probe is positioned to view the mitral valve. By adjusting the C-arm gantry to align with the head of the TEE probe, the EchoNavigator displays a 3-dimensional TEE image in the C-arm view, providing a direct view of the mitral valve [19]. (**b**) Schematics of the proposed training system. (A) Image of a 3D printed heart model on a bi-plane c-arm. (B) Close-up view of a patient-specific 3D printed heart model. (C) Diagram of the process of transferring images and tracking the catheter after processing. (D) Steps involving image processing and deep learning applied to bi-plane images along with a tracking plot [20].

Figure 5 illustrates instances of AR/MR device utilization in interventional cardiology for educational and training objectives.

## 6. Future Perspectives

The incorporation of AR/MR technologies into interventional cardiology holds immense promise for transforming procedural workflows and enhancing patient outcomes. Our previous sections have meticulously examined prior research in this domain. Additionally, Table 1 provides a comprehensive synthesis of these studies, offering a detailed comparison of the key parameters extracted from the literature. Specifically focusing on the utilization of AR/MR technologies in interventional cardiology, these findings encapsulate the current landscape. Despite considerable advancements, pivotal challenges persist, necessitating concerted efforts to fully exploit the potential of these groundbreaking technologies. This section delineates three potential future perspectives poised to influence the trajectory of AR/MR applications in interventional cardiology.

### 6.1. Seamless Integration of Commercial Interventional Tools into AR/MR Visualization

One of the critical challenges in leveraging the benefits of AR/MR visualization for interventional cardiology procedures lies in the seamless integration of standard and commercial interventional tools, such as catheters and guidewires. Currently, many AR/MR systems rely on all virtual representations of these devices or use gamepads instead, which may not accurately replicate the tactile feedback and nuanced movements experienced during actual procedures. Addressing this limitation by enabling direct interaction with standard interventional tools within the AR/MR environment is a crucial step toward enhancing procedural realism and harnessing the full potential of these technologies. This integration would allow interventional cardiologists to experience the familiar tactile sensations and manipulations they are accustomed to while benefiting from the enhanced visualization and guidance provided by AR/MR systems. Initially, this endeavor could focus on simpler catheterization procedures for training purposes, gradually expanding to encompass more complex interventions involving specialized catheters, such as intracardiac echocardiography (ICE) catheters or double-catheter procedures. Ultimately, the goal would be to enable the use of AR/MR visualization during actual surgical procedures, enhancing patient safety and procedural outcomes.

### 6.2. Patient-Specific 3D Heart Visualization through Deep Learning-Driven Segmentation

Accurate and patient-specific 3D visualization of cardiac anatomy is paramount for the effective implementation of AR/MR systems in interventional cardiology. While pre-operative CT scans and manual segmentation techniques have been employed, these processes can be time-consuming and subject to human error. The integration of advanced deep learning-based segmentation algorithms [24,25,26] for automated and rapid 3D heart segmentation could revolutionize this aspect of AR/MR visualization. By leveraging the power of deep learning models trained on vast datasets of cardiac imaging data, it may become feasible to generate accurate, patient-specific 3D heart models in real-time or near real-time. These models could then be seamlessly integrated into the AR/MR environment, enabling clinicians to visualize and co-register the patient’s unique cardiac anatomy with the live positioning of the interventional devices. Currently, these methods primarily use CT and MRI as they provide holistic views of the heart, but these modalities are typically acquired many days before the procedure. Thus, developing methods to produce realistic 4D renderings of the heart from ultrasound or rotational angiography could unlock unprecedented visualization capabilities. The successful development of such deep learning-driven segmentation algorithms would not only streamline the preprocedural preparation process but also enhance the precision and accuracy of AR/MR-guided interventions. Continuous refinement and validation of these algorithms to accommodate 4D imaging in collaboration with clinical experts will be crucial to ensure their reliable performance across a diverse range of patient anatomies and pathologies.

### 6.3. Fluoroscopy-Free Catheterization through Sensor-Embedded Catheters and AR/MR Visualization

Fluoroscopic imaging, while widely utilized in interventional cardiology, exposes both patients and healthcare professionals to ionizing radiation and requires the use of cumbersome lead aprons. To mitigate these concerns, the development of sensor-embedded catheters or thin sensorized sheaths for commercial catheters, combined with AR/MR visualization, could pave the way for fluoroscopy-free catheterization procedures. By incorporating miniaturized sensors or markers into the catheter design, these devices can be accurately tracked and visualized within the AR/MR environment, providing real-time guidance and positioning information without the need for continuous fluoroscopic imaging. This approach not only reduces radiation exposure but also enhances procedural ergonomics by eliminating the need for heavy-lead apparel. Fiber Optic Shape Sensors represent a cutting-edge technology with significant potential for advancing the 3D and full-body tracking of catheters [27]. However, it currently faces limitations in compatibility with sheathes. For instance, Fiber Optic RealShape (FORS) technology [28,29] introduces a fiber optic-based catheter capable of achieving submillimeter precision and offering comprehensive three-dimensional shape tracking. This surpasses the reported precision of alternative navigation and tracking technologies, potentially reducing reliance on fluoroscopy (©2021 Philips Research, Amsterdam, The Netherlands). Alternatively, electromagnetic trackers like FreeNav System [30] leverage specific field generators embedded in the patient beds along with receiving components integrated into catheters to provide a fluoroscopy-free 3D tracking system for catheters. Overcoming the challenges associated with sensor integration, such as biocompatibility, miniaturization, and seamless data transmission, will be critical for the successful implementation of this technology. Collaborative efforts between interventional cardiologists, electrical and mechanical engineers, and material scientists will be essential for developing safe, reliable, and user-friendly sensor-embedded catheter systems. As the field of interventional cardiology continues to evolve, the integration of AR and MR technologies holds immense promise for driving innovation, improving patient outcomes, and enhancing the overall efficiency and safety of complex cardiac interventional procedures.

## 7. Discussion 

This review paper presents a comprehensive and systematic comparative analysis of several AR/MR systems across various key parameters relevant to their applications in interventional cardiology (Table 1). This compilation serves as a useful resource that encapsulates the current state-of-the-art technology in this rapidly evolving field. A striking observation of this study is the diversity of imaging modalities integrated into these AR/MR systems, including computed tomography (CT), magnetic resonance imaging (MRI), fluoroscopy, ultrasound, and electroanatomic mapping systems (EAMS). Among the aforementioned imaging methods, Computed Tomography (CT) and Computed Tomography Angiography (CTA) stand out as the most frequently utilized devices in the literature, being employed in 36% of the reviewed papers. This multifaceted fusion of imaging data highlights the versatility of AR/MR technologies for seamlessly visualizing and combining information from multiple sources, thereby enhancing procedural guidance, anatomical understanding, and decision-making capabilities. 

A significant finding of this analysis is the differential utilization of various extended reality (XR) technologies. Mixed Reality (MR) has emerged as the most prominent, being utilized in 50% of the applications across all examined sections. Augmented Reality (AR) follows with a 41% utilization rate, predominantly employed in education and training contexts. In contrast, Virtual Reality (VR) accounts for just 9% of the usage, indicating its limited application in interventional cardiology. This limited applicability in surgical and clinical settings can be attributed to VR’s inherent characteristic of displaying only a virtual image, which restricts its practical utility in such environments. In these contexts, both AR and MR demonstrate superior effectiveness due to their ability to integrate virtual elements with the real world, enhancing their functionality and applicability in clinical and surgical applications.

The analysis underscores the Microsoft HoloLens as the predominant choice among AR/MR headsets, comprising around 41% of the reviewed systems. This trend underscores the widespread acceptance and applicability of the HoloLens platform in interventional cardiology applications, likely owing to its advanced capabilities, intuitive interface, and compatibility with diverse imaging modalities. However, it is worth acknowledging that while the HoloLens has a legacy status in the VR headset market, newer counterparts like META Quest or Apple Vision Pro, which have been released more recently, may exhibit enhanced functionality in future studies.

Remarkably, the objectives of the reviewed studies span a broad range of clinical applications, encompassing transcatheter aortic valve implantation (TAVI), percutaneous coronary intervention (PCI), electrophysiology mapping and ablation, chronic total occlusion (CTO) revascularization, left atrial appendage (LAA) occlusion planning, and interventions for congenital heart disease. This diversity underscores the versatility and potential impact of AR/MR technologies across various domains of interventional cardiology, positioning them as powerful tools for improving patient outcomes and advancing cardiovascular care.

Notably, a significant portion of the reviewed studies (approximately 24%) focused on the development and validation of AR/MR technologies. These efforts aim to address the challenges related to system performance, image optimization, user interface design, and seamless integration into complex clinical workflows. Continuous refinement and validation of these technologies are crucial for ensuring their reliable and user-friendly implementation in real-world scenarios.

Additionally, several studies (approximately 12%) have explored the educational and training applications of AR/MR technologies in interventional cardiology. These applications leverage immersive and interactive learning experiences to foster skill development, spatial reasoning abilities, and competencies among medical professionals. By simulating realistic procedural scenarios in a risk-free environment, AR/MR technologies have the potential to revolutionize medical education and training, ultimately contributing to enhanced patient care and safety.

This quantitative analysis highlights the diverse applications, technological advancements, and ongoing efforts in integrating AR/MR technologies into interventional cardiology practices. The integration of these cutting-edge technologies holds immense promise in driving innovation, enhancing patient outcomes, and reshaping the future of cardiovascular care by providing unprecedented visualization, guidance, and decision-making capabilities during complex interventional procedures.

## 8. Conclusions

The integration of AR/MR technologies into interventional cardiology represents a transformative frontier, poised to redefine procedural workflows, enhance visualization, and ultimately improve patient outcomes. This review has explored the multifaceted applications of these cutting-edge technologies across four distinct domains: medical AR/MR systems, clinical applications and evaluation, technology development and validation, and educational and training applications. The elucidation of the technological foundations underpinning medical AR and MR systems has shed light on the driving forces behind innovation in this field. Furthermore, the examination of clinical applications has underscored the tangible benefits of these systems in improving procedural precision, efficiency, and decision-making across various cardiac interventions, including transcatheter aortic valve implantation, percutaneous coronary interventions, and electrophysiology mapping and ablation. Notably, the review has delved into the ongoing efforts to refine and validate AR/MR platforms, highlighting the importance of seamless integration into the complex landscape of interventional cardiology procedures. Additionally, the pivotal role of these technologies in medical education and training has been explored, emphasizing their potential to simulate realistic procedural scenarios and foster the acquisition of essential skills among medical professionals.

While significant progress has been made, several challenges remain to be addressed to unlock the full potential of AR/MR technologies in interventional cardiology. These include the seamless integration of standard interventional hardware into AR/MR visualization, the development of patient-specific 3D heart visualization through deep learning-driven segmentation, and the pursuit of fluoroscopy-free catheterization through sensor-embedded catheters and AR/MR visualization. Overcoming these challenges will require collaborative efforts among interventional cardiologists, biomedical engineers, material scientists, and researchers, as well as continuous refinement and validation of these technologies. By addressing these challenges, the field of interventional cardiology can harness the transformative power of AR and MR technologies, drive innovation, enhance patient safety, and optimize procedural outcomes. As the healthcare landscape continues to evolve, the integration of AR and MR technologies into interventional cardiology practices holds immense promise for advancing the field toward more effective, efficient, and patient-centric cardiovascular care. Through continuous research, development, and validation, these innovative technologies will undoubtedly play a pivotal role in shaping the future of interventional cardiology and improving the lives of patients worldwide.

## Figures and Tables

**Table 1:** Summary and comparative analysis of the reviewed AR/MR systems across key parameters.

Ref.	AR/MR/VR	Headset	Imaging System/Technology	Related Section	Application	Novelty and Advantage	Year
[14]	VR/AR	Head-mounted display	CT	Technology Development and Validation	Minimally invasive cardiac procedures	Surgical guidance evaluation	2010
[19]	VR/AR	Echo Navigator	RA	Educational and Training Applications	Congenital heart disease Catheterization	3D imaging technologies in interventional	2014
[11]	VR/AR	RealView Holographic Display	3DRA	Clinical Application and Evaluation of AR/MR Systems	Anatomical landmarks, Usability of interactions, Standard catheterization laboratory	Real-time interactive 3D digital holograms	2016
[12]	VR	Google glasses	CTA	Clinical Application and Evaluation of AR/MR Systems	Percutaneous revascularization and coronary chronic total occlusion	CT guided Wearable computer	2016
[2]	VR	zSpace 200, polarized glasses	2D medical scan images (such as CT)	Medical AR/MR Systems	Blood simulation	Decision support in cardiovascular events	2017
[21]	AR	Google glasses	CTA	Clinical Application and Evaluation of AR/MR Systems	Coronary chronic total occlusion	Feasibility and safety of CT-assisted percutaneous revascularization	2017
[18]	AR	Smart glasses (BT-350)	CT	Educational and Training Applications	Cannula guide system	Blood vessel and deep venous vessel images	2018
[23]	AR	Google glasses	CTA	Educational and Training Applications	Transcatheter pacemaker implantation	Congenitally corrected transposition and fluoroscopic orientation	2018
[15]	AR	Microsoft HoloLens	X-ray fluoroscopy, pre-operative CT	Technology Development and Validation	Transcatheter procedures and Structural heart disease	3D holographic rendering and Image guidance	2019
[17]	MR	Microsoft HoloLens	CTA	Technology Development and Validation	Congenital heart disease	3D heart models and Pre-operative diagnostic	2019
[4]	MR	Microsoft HoloLens	EnSite^TM^ Velocity^TM^	Medical AR/MR Systems	Catheter locationing	3D images for Electroanatomic mapping procedures	2020
[9]	MR	Microsoft HoloLens	EAMS	Clinical Application and Evaluation of AR/MR Systems	Minimally invasive surgery	Intraprocedural hands-free dynamic control	2020
[1]	MR	Microsoft HoloLens	MRI	Medical AR/MR Systems	MRI of the beating heart	Online with spatiotemporal resolutions	2021
[20]	MR	---	X-ray fluoroscopy	Educational and Training Applications	Catheter tracking and minimally invasive surgery	Deep learning, 3D printing	2021
[3]	MR	MR head-mounted display (MR-HMD)	Real-time fluoroscopy, Vascular ultrasound	Medical AR/MR Systems	Right heart catheterization	Wire fluoroscopy, Vascular ultrasound, Optimize procedure time	2022
[5]	MR	HMD	CT	Medical AR/MR Systems	Left atrial appendage occlusion planning	3D visualization, Accurate and fast planning phase	2022
[8]	MR	Microsoft HoloLens	ultrasound image	Clinical Application and Evaluation of AR/MR Systems	Active guidance of needle repositions	evaluating the quality of access, an ultrasound system	2022
[10]	MR	Microsoft HoloLens v2	Ultrasound-guided puncture	Clinical Application and Evaluation of AR/MR Systems	vascular puncture guidance	TAVI, real-time ultrasound, CT under aseptic conditions	2022
[16]	AR	Microsoft HoloLens v2	Electromagnetic sensors	Technology Development and Validation	Catheter tracking and Radiation-free intervention	Electromagnetic sensors	2022
[6]	MR	Microsoft HoloLens v2	Ultrasound scanner	Medical AR/MR Systems	Ultrasound-guided femoral arterial cannulations	Enabling real-time practice of interventional cardiology	2023
[7]	AR	Microsoft HoloLens v2	3DRA	Medical AR/MR Systems	Congenital heart disease	Educational potential	2023
[13]	MR	Microsoft HoloLens v1	EAMS	Technology Development and Validation	Cardiac electrophysiological testing	Reduced procedural times and case duration	2023

## Data Availability

No new data were created or analyzed in this study. Data sharing is not applicable to this article.
